# Modern Gynecology

**Published:** 1899-01

**Authors:** 


MODERN GYNECOLOGY.—A Treatise on Diseases of Women, comprising the results of the latest investigation and treatment in this branch of medical science. By Chas. H. Bushong, M. D., Assistant Gynecologist to the Demilt Dispensary, New York, formerly Attending Physician to the Northern Dispensary, New York. Illustrated. Second edition, enlarged. Published by E. B. Treat & Co., New York. Pages, 400. 1898. Price, cloth, $2.00

A fairly complete and accurate statement of present gynecological practice, addressed especially to the necessities of the busy general practitioner, and will prove of value to him, by presenting in brief, concise terms the latest improvements in technique and practice. The literary style is classical in its perfection and simplicity.

I cannot agree to at least one of the procedures recommended, however, viz.: the reposition of a misplaced uterus with the uterine sound or repositor. I observe that this proceeding is recommended, too, without the slightest word of caution against pushing the sound through a diseased uterus into the abdominal cavity. This caution was formerly never omitted in standard text books. In my judgment the danger I have referred to is very real, in fact, it has occurred in my own practice in spite of the caution used. I do not believe the procedure is ever really indicated. If the malposition is caused by simple engorgement of the uterus, and it is desired to replace it, it can always be done either by bimanual manipulation or by placing the patient in the knee-breast position and balooning the vagina. If it is bound down by adhesions it cannot be replaced until they are broken up, and the attempt to replace with the sound in this condition is extremely dangerous. Therefore, the use of the sound for this purpose is a useless and dangerous procedure in any event. With this omission, and others of minor degree excluded, the work is a very creditable and useful one. I. J. J.

